# Decreased hemoglobin as a quantifiable indicator of renal arterial embolization in post-percutaneous nephrolithotomy hemorrhage

**DOI:** 10.1007/s00240-020-01206-6

**Published:** 2020-08-08

**Authors:** Ruitu Ran, Ruiyuan Zhang, Ye Xie, Zhikang Yin

**Affiliations:** 1 Departments of Urinary Surgery, The First Affiliated Hospital of Chongqing Medical University, No. 1 Youyi Road, Yuzhong District, Chongqing, 400016 People’s Republic of China; 2 Department of Occupational and Environmental Health, School of Public Health and Management, Research Center for Medicine and Social Development, Innovation Center for Social Risk Governance in Health, Chongqing Medical University, Chongqing, People’s Republic of China

**Keywords:** Percutaneous nephrolithotomy, Hemorrhage, Embolization, Hemoglobin

## Abstract

To determine quantifiable indicators for post-percutaneous nephrolithotomy (PCNL) renal arterial embolization. A total of 2043 patients who underwent PCNL from September 2012 to March 2018 were reviewed retrospectively. Post-operative hemorrhage patients were extracted and divided into two groups according to treatment methods (conservative methods or super-selective renal arterial embolization [SRAE]). Demographic characteristics and hemorrhage outcomes were compared between the two groups by univariable analysis. Multivariable logistic regression was used to reveal the association between hemorrhage outcome factors and SRAE. A receiver operating characteristic (ROC) curve was drawn to determine the optimized cut-off value for SRAE. We identified 71 patients who had post-PCNL hemorrhage. Seventeen and 54 patients comprised the SRAE and conservative groups, respectively. No significant differences in demographic characteristics were found between the two groups. Univariate analysis showed that the differences in decreased hemoglobin (Hb), hemorrhage types, and transfusion were significant between the two groups (*p *< 0.001). Multivariable analysis showed that the decreased Hb was closely associated with the risk of SRAE. The ROC curve showed that an adjusted Hb decrease of 3.45 g/dL was an optimum indicator (AUC = 0.925). Decreased Hb is an indicator for SRAE after PCNL. When the adjusted decrease in Hb is ≥ 3.45 g/dL, SRAE should be performed regardless of the manifestations of hemorrhage.

## Introduction

Percutaneous nephrolithotomy (PCNL), the first-line treatment for large or complex renal stones, was initially reported by Fernstrom and Johansson in 1976 [1]. This procedure offers high stone-free rates and a short recovery time [2]; however, post-PCNL complications are not completely avoidable [3]. One of the most common and severe complications is hemorrhage. Transfusion and embolization rates with different indications after PCNL are 0.7%–55% and 0.6%–1.5%, respectively [4–6].

Selective renal angiographic embolization (SRAE) is a high-efficiency, mini-invasive procedure which is recognized as the treatment of choice for post-PCNL hemorrhage. The rates of hemorrhage cessation via SRAE have been reported to be > 90% [7–9]; however, SRAE may induce corresponding complications, such as artery injury and decreased renal function. In different clinical conditions, the indication for angioembolization can be different. Hemodynamic instability after conservative management is an accepted indication for renal angiography in post-PCNL hemorrhage, but most patients with hemorrhage are hemodynamically stable [10]. For hemodynamically stable patients, the severity of bleeding can be an indication for angioembolization. A decrease in hemoglobin (Hb) is the most widely used index for determining the severity of bleeding [11–14]. Several studies have proposed that an Hb decrease > 3 g/dL while undergoing transfusions is an accepted indication for angioembolization in different surgical conditions [11–14], but there are few studies which have focused on establishing quantified indications for SRAE in the setting of post-PCNL hemorrhage.

In the present study, univariable and multivariable analyses of clinical characteristics within the conservative and embolization groups were performed to provide a quantified indication for SRAE in patients with post-PCNL hemorrhage.

## Patients and methods

This study was approved by the First Affiliated Hospital of Chongqing Medical University hospital Institutional Review Board (Chongqing, China). We retrospectively reviewed 2043 patients who underwent PCNL surgery at our hospital from September 2012 to March 2018. The study inclusion criteria were post-PCNL hemorrhage. The exclusion criteria were a history of blood disorders and perioperative anti-platelet agent use. Seventy-one patients were included in our study based on the above criteria and were divided into two groups according to treatment methods (conservative group[n = 54] and SRAE group [n = 17]), and all patients with hemorrhage included in our study were hemodynamically stable. Two patients with hemorrhage and unstable hemodynamics who underwent open nephrostomies immediately as life-saving procedures were not included in our study. No deaths were reported in our study.

All PCNLs were performed by two experienced surgeons. Patients were placed in the prone position under general anesthesia. Percutaneous punctures were performed with ultrasound guidance, and the tracts were dilated to 18–26 Fr with Amplatz. The number and location of tracts depended on the stone characteristics and anatomic variables. Holmium laser or ultrasonic lithotripters were used to remove stones. Nephrostomy tubes were retained at the end of the procedure and ordinarily removed after 3–5 days of PCNL. In addition, the tube was retained for a longer period if hemorrhage and fever occurred after PCNL. Serial Hb tests were performed from the first post-operative day. The endpoint of serum Hb observation was no recurrent bleeding after PCNL, including hematuria, bloody nephrostomy drainage, retroperitoneal bleeding, hemodynamic instability, and fluctuations in the Hb level.

Patients with hemorrhage were initially managed with conservative measures, such as bed rest, hemostatic drugs, adequate hydration, tamponade by clamping the nephrostomy tube, and transfusion. The indication for transfusion was a Hb level < 7 g/dL, and the volume of a 1-unit blood transfusion was 400 ml. If conservative management was ineffective, SRAE was sequentially performed to control the bleeding.

**Fig. 1:**
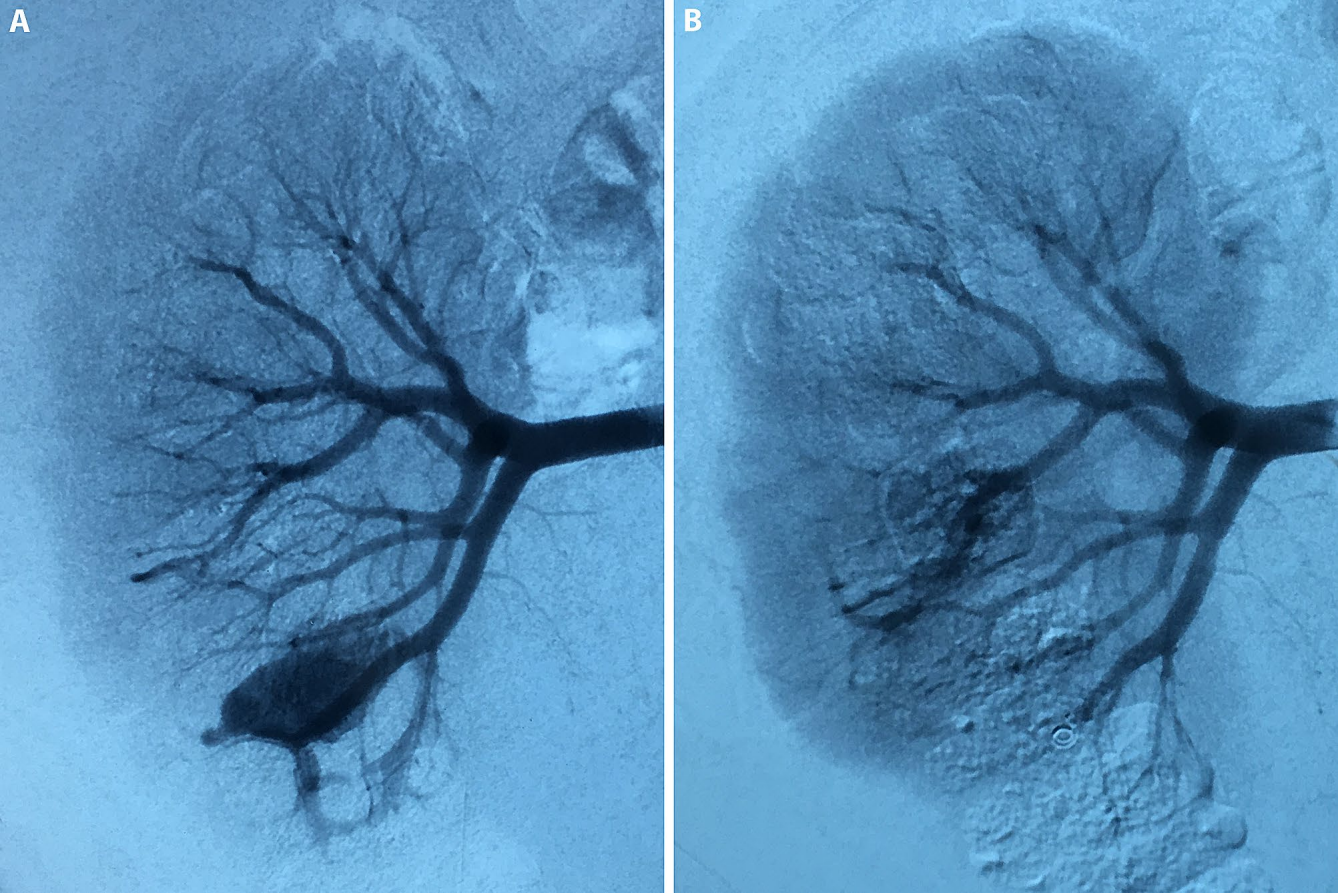
SRAE was performed to control hemorrhage. **a** The pseudoaneurysm and arteriovenous fistula originated from the inferior segmental artery, which was identified by right renal artery angiography. **b** The injured vessel was selectively embolized by the coils, and the angiogram was repeated to assess the outcomes of hemostasis

SRAE was performed via the transfemoral approach under local anesthesia. Renal arteriography was performed with a C2 catheter and super smooth guidewire, which was used to enter the renal artery. Depending on the angiographic findings, a 3-Fr coaxial microcatheter was used to access the bleeding vessels, then appropriate coils were chosen to occlude the bleeding point. Finally, the renal angiogram was repeated to confirm that bleeding was completely controlled (Fig. 1). Patients were discharged from the hospital when the clinical judgment of the hemorrhage cessation was ensured. All patients were followed for 6–12 months and no recurrences of hemorrhage occurred.

Data collected from the medical records included age, gender, body mass index (BMI), involved side, stone burden and size, severe hydronephrosis, diabetes mellitus, hypertension, liver dysfunction, disease course, past ipsilateral kidney intervention (open surgery, PCNL, and extracorporeal shock wave lithotripsy [SWL]), number and size of tracts, operative time, and urinary tract infection. Hemorrhage outcomes included pre-operative Hb levels, decrease in Hb, transfusion rate and number of units, and hemorrhage time and types. Hemorrhage time is described by days from the beginning of bleeding to cessation. The manifestations of hemorrhage provide the visual and intuitional information for surgeons to make an appropriate judgment on treatment choice. Yet, standard classifications on post-PCNL hemorrhage do not exist. To adequately analyze the relationship between the hemorrhage features and embolization, we classified all hemorrhage patients into three different types based on clinical manifestations and existing criteria from previous research [10], as follows: type 1, sudden onset of hemorrhage (patients suddenly experienced bright-red hematuria or bloody nephrostomy drainage with clots and the amount of bleeding was large.); type 2, intermittent hemorrhage (patients presented with a recurrence of hematuria or nephrostomy drainage and sporadic blood clots are found in the drainage bag. The color of drainage can be crimson or dark red, and the time between two hemorrhage episodes may last for several hours or days); and type 3, continuous chronic hemorrhage (with red urine or drainage, blood clots usually cannot be found in the drainage bag. The hemorrhage time can last from several days to weeks).

Decreased Hb is calculated by the highest Hb minus the lowest Hb within the observation time of serial Hb tests. The decrease in Hb was corrected by the number of transfusions. One unit of packed red blood cells or whole blood transfusion was equal to a 1.0 g/dL decrease in Hb.

Data are expressed as the mean ± standard deviation (SD). The univariate analyses were performed to analyze demographic data and hemorrhage outcomes with Chi squared and non-parametric tests. Multivariable logistic regression was used to explore the association between the hemorrhage factors and SRAE. A receiver operating characteristic (ROC) curve was drawn to determine the optimized cut-off point of decreased Hb for SRAE, hence the decreased Hb data for all the hemorrhage patients were plotted in the ROC curve to determine the best cut-off point. Statistical analyses were performed using SPSS 23.0 software and a *p *< 0.05 was statistically significant.

## Results

Seventy-one patients who met the study inclusion criteria were included in the present study. All the hemorrhage patients were divided into two groups (conservative and SRAE groups). Seventeen and 54 patients comprised the SRAE and conservative groups, respectively. At baseline, the mean age of the conservative group was 50.3 ± 15.4 years; the SRAE group was 51.5 ± 9.1 years. The patients included in the conservative group were successfully managed with conservative methods, and the hemorrhage patients that did not respond to the conservative methods were included in the SRAE group. The success rate of SRAE was 94.1% because one patient failed SRAE and underwent open surgery to stop the bleeding. Among all of the patients, only one patient had a renal abnormality (a horseshoe kidney) and underwent conservative management.

As shown in Table 1, baseline characteristics of demographic data showed no significant statistical differences between the two groups.

**Table 1:** Baseline characteristics

Characteristics		Conservative group (*n *= 54)	SRAE group (n = 17)	*p* value
		*n* (%)	*n* (%)	
Sex	Female	12 (22.2%)	2(11.8%)	0.551
	Male	42 (77.8%)	15(88.2%)	
Age(y)	≤ 30	7 (13.0%)	0	0.860
	30–60	32 (59.3%)	14(82.4%)	
	> 60	15 (27.8%)	3(17.6%)	
BMI(kg/m^2^)	≤ 18.4	8 (14.8%)	1(5.9%)	0.181
	18.5–23.9	29 (53.7%)	8(47.1%)	
	≥ 24	17 (31.5%)	8(47.1%)	
Hypertension	Yes	9 (16.7%)	4(23.5%)	0.781
	No	45 (83.3%)	13(76.5%)	
Diabetes	Yes	4 (7.4%)	4(23.5%)	0.163
	No	50 (92.6%)	13(76.5%)	
Liver dysfunction	Yes	0	1(5.9%)	0.239
	No	54 (100.0%)	16(94.1%)	
Involved Side	Right	31 (57.4%)	10(58.8%)	0.918
	Left	23 (42.6%)	7(41.2%)	
Stone burden	Multiple calculi	20 (37.0%)	9(52.9%)	0.492
	Staghorn calculi	10 (18.5%)	3(17.6%)	
Severe Hydronephrosis	No	52 (96.3%)	14(82.4%)	0.157
	Yes	2 (3.7%)	3(17.6%)	
Disease course(y)	< 10	30 (55.6%)	4(23.5%)	0.297
	≥ 10	24 (44.4%)	13(76.5%)	
Past ipsilateral kidney intervention	Open surgery	9 (16.7%)	1(5.9%)	0.236
	SWL	4 (7.4%)	3(17.6%)	
	PCNL	2 (3.7%)	2(11.8%)	
Size of tracts	18 F	21 (38.9%)	9(52.9%)	0.152
	24 F	27 (50.0%)	8(47.1%)	
	26 F	6 (11.1%)	0	
Number of tracts	1	52 (96.3%)	16(94.1%)	0.566
	≥ 2	2 (3.7%)	1(5.9%)	
Operation time	< 100 min	29 (53.7%)	8(47.1%)	0.632
	≥ 100 min	25 (46.3%)	9(52.9%)	
Urinary infection	Yes	34 (63.0%)	8(47.1%)	0.245
	No	20 (37.0%)	9(52.9%)	
Blood transfusion	Yes	9 (16.7%)	12(70.6%)	< 0.001
	No	45 (83.3%)	5(29.4%)	
Hemorrhage type	Type1	12 (22.2%)	9(52.9%)	0.036
	Type2	25 (46.3%)	5(29.4%)	
	Type3	17 (31.5%)	3(17.6%)	

Significant statistical differences existed in terms of blood transfusion (*p *< 0.001) and hemorrhage type (*p *= 0.036) between the two groups (Table 1). Type 1 hemorrhage accounted for the largest proportion in the SRAE group (52.9%), accounting for the lowest proportion in the conservative group (22.2%). Type 2 and 3 hemorrhage together accounted for 47.0% in the SRAE group and 77.8% in the conservative group (Table 1). The comparison showed the proportion of types was significantly different between the two groups. The hemorrhage features between the three types of hemorrhage were analyzed to verify that the classification was rational. As shown in Table 2, the hemorrhage time of type 3 hemorrhage (8.9 ± 5.20 d) was longer than the time for type 1 hemorrhage (4.05 ± 1.50 d, *p *< 0.001). The decreased Hb (3.95 ± 1.94 g/dL) in type 1 hemorrhage had a greater degree of decline than the decrease in type 2 (1.94 ± 1.56 g/dL) and in type 3 hemorrhage (2.47 ± 1.37 g/dL; *p* = 0.001). The hemorrhage time and decreased Hb was distinctive among the three types of hemorrhage. The differences in decreased Hb (*p *= 0.001) and hemorrhage time (*p *< 0.001) among the three types of hemorrhage were significant and the results are shown in Table 2.

**Table 2:** Hemorrhage characteristics in the 3 types of hemorrhage

	Type 1	Type 2	Type 3	*p* value
No. patients(%)	21 (29.6%)	30 (42.3%)	20 (28.2%)	
Preoperative hemoglobin level(g/dL)	13.57 ± 1.56	13.38 ± 2.00	13.70 ± 1.61	0.788
Hemorrhage time(day)	4.05 ± 1.50	7.6 ± 5.33	8.9 ± 5.20	< 0.001
Decreased Hb (g/dL)	3.95 ± 1.94	1.94 ± 1.56	2.47 ± 1.37	0.001
Transfusion rate(%)	9 (42.9%)	8 (26.7%)	4 (20%)	0.249
Transfusion volume(unit)	2.72 ± 1.55	2.41 ± 1.86	1.50 ± 0.87	0.438

Next, a comparison in terms of the pre-operative Hb level, decreased Hb, number of units transfused, and hemorrhage time between the SRAE and conservative groups showed that the SRAE group had a greater decrease in Hb (4.83 ± 1.51 g/dL, *p *< 0.001, Table 3). Additionally, there was a trend toward a longer hemorrhage time in the SRAE group (9.39 ± 7.95 d), but the difference was not statistically significant (*p *= 0.142). The decreased Hb, hemorrhage time, and units of blood transfused were added to multivariate logistic analysis (Table 4). The results revealed that the decreased Hb was the only factor which was closely related to embolization. When the decreased Hb was > 2.1 g/dL, a 1 g/dL decrease in the Hb level was associated with a 2.739-fold increase in the risk of post-PCNL SRAE (*p *= 0.001). The results of multivariable analysis showed that neither the hemorrhage time nor the number of units transfused was associated with SRAE.

**Table 3:** Characteristics of hemorrhage outcomes (mean ± SD)

	Conservative group	SRAE group	*p* value
Preoperative Hb level (g/dL)	13.54 ± 1.85	13.42 ± 1.50	0.807
Hemorrhage time (day)	6.15 ± 2.86	9.39 ± 7.95	0.142
Decreased Hb (g/dL)	2.01 ± 1.40	4.83 ± 1.51	<0.001
Transfusion volume (unit)	2.03 ± 1.57	2.62 ± 1.93	0.734

**Table 4:** Association between characteristics and SRAE outcomes

	β Coefficient	Adjusted OR(95% CI)^a^	*p* value
Blood transfusion	0.281	1.324(0.214-8.193)	0.763
Decreased Hb (g/dL)	1.008	2.739(1.532-4.896)	0.001
Hemorrhage time(day)	0.148	1.159(0.961-1.398)	0.123

^a^Adjusted for all variables in this table

**Fig. 2:**
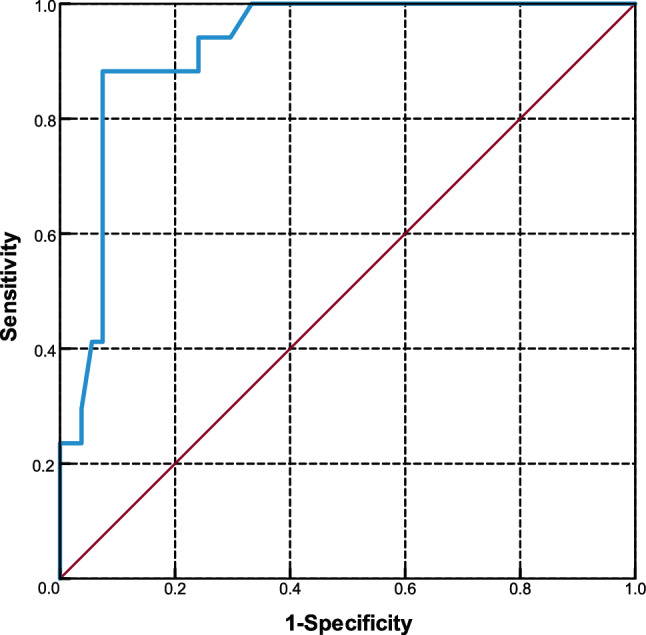
ROC curve of decreased Hb in 71 PCNL patients with 54 conservative treatment patients as a control. The accuracy, cut-off, sensitivity, and specificity were 92.5%, 3.45, 88.2%, and 92.6%, respectively

To detect the most appropriate cut-off point for decreased Hb, a ROC curve was drawn and showed the sensitivity (88.2%) and specificity (92.6%) for the decreased Hb. We found that the best cut-off point was 3.45 g/dL (*p *< 0.001, Fig. 2). A decrease in Hb ≥ 3.45 g/dL was a significant indicator for post-PCNL SRAE.

## Discussion

With the rising incidence and prevalence in urinary stones, PCNL is increasing worldwide in recent decades due to the advantages of the mini-invasive procedure and short recovery period [15, 16]. Additionally, PCNL has high stone-free rates, which could be a reasonable choice for patients with large and/or complex upper urinary tract calculi. PCNL, however, can cause complications, including hemorrhage, fever, urinary infection, renal colic, and septicemia similar to an invasive procedure. Hemorrhage is one of the most frequent post-PCNL complications and is usually caused after the rupture of parenchymal vessels, which can arise from arterial and venous lesions [8, 17]. Fortunately, most venous hemorrhage can be controlled conservatively. The massive hemorrhage that requires transfusion or intervention normally induced by the injury of the segmental arteries or secondary arterial branches [8].

Hemorrhage is classified into different types based on various criteria. According to the different level of hematuria, Srivastava et al. [18] classified post-PCNL hemorrhage into 3 categories (mild, moderate, and severe), but there no explanation of the classification scheme in their report. Kessaris et al. [19] classified the hemorrhage according to the occurrence time of hematuria, as follows: I. hematuria occurring during percutaneous surgery; II. hematuria occurring at nephrostomy tube removal; and III. hematuria occurring more than 7 days after percutaneous surgery. The above studies did not integrate the hemorrhage time and amount of bleeding sufficiently; hence, the indicators that were proposed were not sufficiently comprehensive. Li et al. [10] classified post-PCNL hemorrhage into three types (sudden onset bleeding, intermittent bleeding, and continuous slow bleeding), which gave a more detailed description of post-PCNL hemorrhage. Based on the presentation of patients and the classification that previous study had proposed, we separated hemorrhage into three types, which is similar to the Li et al. study [10]. Previous studies did not validate the rationality of the classification. In our study, we analyzed the hemorrhage factors between three types of hemorrhage. The results showed that the hemorrhage time and decreased Hb were significantly different, revealing that the classification is reasonable and some type-2 and 3 cases experienced arduous conservative treatment, and finally SRAE to stop the hemorrhage. In these cases, hemorrhage time was obviously prolonged, thus increasing the physical and psychological burden for patients, and creating a dilemma for surgeons (i.e., whether or when to perform SRAE).

SRAE is a valuable treatment for most renal vascular injuries [20]. Compared with conservative methods, SRAE is highly effective and mini-invasive [21, 22]. SRAE also may cause a series of corresponding complications, such as arterial dissection, arterial perforation, contrast-induced nephropathy, and loss of renal parenchyma [22, 23]. Previous studies have reported the indications for SRAE. Kumar and colleagues [24] proposed that renal angiography should be performed when the hemorrhage was not responsive to conservative maneuvers or there was massive hematuria with a decrease in Hb by 2 g/dl or a transfusion of ≥ 4 units. Kessaris et al. [19] pointed out that hemorrhage occurring 2–7 days after PCNL required a 3–4 unit blood transfusion, and hemorrhage occurring beyond 7 days post-PCNL should be treated with angiography and embolization. Jain et al. [25] illustrated the absolute indications for angioembolization as follows: (1) hemodynamic instability due to life-threatening bleeding; (2) repeated clot evacuation; (3) repeated blood transfusions; (4) renal Doppler showing vascular lesions; and (5) continued decline in Hb. Bleeding presenting with hemodynamic instability is an accepted indication for post-PCNL embolization, but there are still no uniform indicators for hemodynamic instability. Moreover, the indications mentioned above lack specific indices and have not been validated.

Decreased Hb is the most widely used laboratory marker for the severity of bleeding. In hemodynamically stable patients, the severity of bleeding can be an indication for embolization in different surgical conditions. Therefore, decreased Hb was regarded as a useful indicator of embolization [11–14]. The indicator, decreased Hb, has rarely been reported in series of post-PCNL hemorrhage; a literature review revealed only one article that quantified the indicator for SRAE in hemorrhage after PCNL [10]. Li and colleagues [10] conducted a multi-center retrospective study which involved 17,630 PCNL patients and proposed that a corrected Hb decrease > 3 g/dL following conservative management is an indication for angiographic embolization while they did not include a conservative group and the selected cut-off point was the minimum value of decreased Hb among the embolization cases.

In the present study, the multivariate analysis of risks for SRAE showed that for a decreased Hb > 2.1 g/dL, a 1 g/dL decrease in Hb was associated with a 2.739-fold increase in the risk of SRAE after PCNL. The results revealed that the decreased Hb had a significant positive correlation with SRAE, which verified the viewpoints of previous studies that decreased Hb was regarded as an indicator for embolization [10, 24]. Besides, we noted that no association existed between transfusion and SRAE, which may be due to the decreased Hb adjusted by the number of units transfused. Considering the specificity and sensitivity of this indicator, the ROC curve was drawn and further analysis of the ROC curve showed that an adjusted Hb decrease of 3.45 g/dL was the most applicable cut-off point for SRAE.

The present study was limited by the small sample size and retrospective design. Multicenter and larger sample studies are required to repeat and verify our results. The hemorrhage types are classified subjectively based on the judgment of researchers, which may introduce a selection bias. To minimize these biases, the assessment process was performed by three individuals with relevant experience. Furthermore, the limitations of this study include the lack of the uniform standard for performing embolization.

## Conclusion

The results of the present study reveal a positive correlation between decreased Hb and the risk of SRAE. When the Hb level was decreased by 2.1 g/dL, the risk of SRAE increased 2.739-fold with a 1.0 g/dL decrease in Hb. Our research, to a certain extent, verified the viewpoint that decreased Hb is an indicator of SRAE in post-PCNL hemorrhage. The patients with hemorrhage and decreased Hb ≥ 3.45 g/dL should proceed the SRAE regardless of the manifestation of hemorrhage. Additionally, we provided a sufficient analysis structure; however, future studies with larger samples in different regions are required to establish more precise and universal indications.
